# Analyzing big tobacco’s global youth marketing strategies and factors influencing smoking initiation by Nigeria youths using the theory of triadic influence

**DOI:** 10.1186/s12889-020-8451-0

**Published:** 2020-03-20

**Authors:** Ukoabasi Isip, John Calvert

**Affiliations:** Faculty of Health Sciences, Simon Fraser University, Burnaby, BC Canada

**Keywords:** Nigeria, Tobacco control, Theory of triadic influence, Youth

## Abstract

**Background:**

Major transnational tobacco companies (TTCs) have identified Nigeria, the seventh most populous country in the world, as a market with a significant revenue potential, given its high youth population and growing gross domestic product (GDP). This research analyses tobacco industry-related strategies and activities targeting youth (aged 15 to 24 years) in Nigeria involving existing, but most importantly, future tobacco users. Nigeria is the focus of this study because the tobacco industry has viewed it as a major emerging market since the 1990s. Successful marketing in Nigeria could provide the industry with a template for similar initiatives in other emerging markets in low- and middle-income countries.

**Methods:**

The research began with a systematic review of secondary literature to determine how the tobacco industry has targeted youth globally and factors contributing to youth smoking initiation. It then used the theory of triadic influence as a heuristic framework to categorize the various factors influencing youth smoking initiation and industry strategies related to increasing tobacco use among youths. Quotations from internal tobacco industry documents were organized into the three streams of the theory of triadic influence: biology/personality, social and cultural/environmental streams. A total of 12 interviews were conducted with 6 policymakers and governmental officials, 2 civil society organization representatives, a high school principal, a journalist and 2 researchers to investigate how the tobacco industry had targeted youth in Nigeria.

**Results:**

The findings indicate that TTCs have actively targeted youth in Nigeria since the 1990s, focusing on changing behaviour through the biology/personality, social and environmental/ cultural streams.

**Conclusion:**

The study suggests that Nigeria implement and vigorously enforce its 2015 National Tobacco Control Bill as well as a package of other measures to prevent tobacco companies targeting youth.

## Background

Tobacco use remains the highest preventable cause of death worldwide today, causing about eight million deaths, annually [45]. Approximately 50% of regular smokers will eventually die from tobacco-related diseases. The World Health Organization (WHO) predicts that 80% of tobacco-related deaths will occur in LMICs by 2030 [44]. This prediction is due to the shift by transnational tobacco companies (TTCs) to emerging markets to compensate for declining sales in traditional (largely high income) markets [23, 26]. There is extensive evidence that TTCs have also pursued new consumers, notably among youth [43]. Euromonitor [15] observes that Nigeria, Indonesia, Mexico, Philippines and Turkey [NIMPT] with growing economies, rising incomes, young and increasing populations, provide major opportunities for TTCs facing reduced demand in developed markets [15].

The Nigerian federal government has affirmed its commitment to introduce new measures to protect the health of the country’s youth. These include a ban on the sale of cigarettes to individuals below the age of 18 years, curbs on advertising aimed at young people, and restrictions on tobacco use at facilities patronised by children such as playgrounds, schools, cinemas and hospitals. It has adopted some of these measures in the 2015 National Tobacco Control Act [18]. But there are major gaps in its approach. Euromonitor International [16] has observed that despite the age requirement, the practice of sending juveniles to buy cigarettes for adult family members is widespread and encourages early exposure to cigarettes, making it difficult to implement age restrictions, thus increasing juvenile smoking rates in Nigeria.

It is this context that makes Nigeria an especially useful site to better understand and develop recommendations for strengthening measures to prevent youth being targeted by the tobacco industry. This research analyses factors influencing youth smoking and industry-related strategies targeting youth (aged 15 to 24 years) in Nigeria as existing, and, future tobacco users. Nigeria is the focus of this research because the tobacco industry has viewed it since the 1990s as a major emerging market [15].

### Theory of triadic influence

In 1999, Flay applied the theory of triadic influence as a heuristic tool to summarise existing knowledge about youth and tobacco, and analyze demand and supply-side measures to address the problem [20]. He concluded that factors such as advertising/promotion, price and access policies are strong influences on youth initiation of tobacco use. He recommended counter-advertising by exposing the harmful effects of tobacco use using various media as a means of reducing tobacco use among young people [21].

This research study applies the theory of triadic influence to classify factors influencing youth smoking and the types of strategies used by the tobacco industry in Nigeria to target youth. This is not to imply that the tobacco industry has consciously operated according to this theory. Rather, this paper uses the theory as a heuristic device to identify the factors influencing youth smoking and the types of industry strategies used to enhance these factors. This framework is applied to categorize the various tobacco industry activities identified through the tobacco industry documents (TIDs) - a large volume of material made public through victim lawsuits - according to three streams of influence, namely; Biology/Personality, Social, and Cultural Environment [19].

#### Three streams of the theory of triadic influence

1. *Biology/personality stream:* The biological/personality stream of the theory of triadic influence concerns personality traits and individual characteristics that provide internal motivation to smoke or increase susceptibility to cigarette smoking addiction. These include the ability to resist pressures to smoke (refusal skills), susceptibility to nicotine addiction and the ability to control the smoking habit (self-efficacy) [20]. These abilities may result from a combination of personality differences, genetic makeup, hormonal differences (males versus females), and other biological/personality factors. For instance, previous research has shown that males are more open to risk-taking behaviours, while females are more open to sensation-seeking behaviour [20].
2. *Social stream:* The social stream concerns social support systems of the adolescent smoker, emotional attachments to important others (parents, siblings, friends), perceptions about normative nature of smoking and pressures to smoke from important others [20].
3. *Cultural/environment stream:* The cultural/environment stream concerns the role of social institutions, government and school policies in shaping societal norms concerning tobacco use, and an individual’s beliefs and evaluation of the costs and benefits of tobacco use [20].

### Categorisation of industry marketing strategies

The literature suggests that another useful way of classifying industry strategies, is by distinguishing between “above the line” and “below the line” marketing [9, 33, 37]. This distinction, used in the marketing field, is found extensively in internal tobacco industry documents. Above the line refers to the use of mass media to promote brands and reach target consumers. These include conventional media such as broadcast and print media, billboards, film, as well as the internet. Below the line refers to alternative marketing methods which use non-mainstream media to focus its direct targeting of consumers instead of targeting a mass audience [9]. Examples include direct mail campaigns, competitions, trade shows and catalogs, and targeted search engine marketing. The youth market has been subtly identified in many TIDs as YAUS (Young adult urban smokers). This designation was most likely to hide strategies targeted at youth [7, 8, 24].

## Methods

### Design

This is a qualitative study which sought to explore and analyze themes related to tobacco industry marketing to youth using the theory of triadic influence as a heuristic framework. The study was conducted as part of the master’s research of the first author.

### Sources of data

Options for data collection were determined by filling gaps in the literature from the paucity of studies on the issue; the lack of tobacco monitoring and reporting by government or civil society organizations; and evidence of the unethical, and, arguably, illegal nature of the tobacco industry’s practices targeting young smokers. Given these challenges, the three main sources of data chosen were: a systematic review of the secondary literature; tobacco industry documents (TIDs); and, semi-structured interviews with key policymakers, civil society organizations and researchers with interests in tobacco control. Inteview data collected lacked the required depth for a thorough analysis on the state of tobacco control in Nigeria due to limited knowledge shown by some of the participants who held various positions related to tobacco control. As a result, interviews only served as additional support to results from the literature review and TIDs. A detailed analysis of the interview results is therefore not presented in this paper. The first author carried out all primary and secondary data collection.

### Review of secondary literature

The purpose of the systematic review of the secondary literature was to identify existing knowledge about the tobacco industry in Nigeria and, in particular, its strategies and activities for targeting the youth. This involved a search of EBSCO Host’s databases and Medline (with full text) and Business Source Complete to identify tobacco industry strategies used to target youth, globally, with a specific focus on LMICs because of their higher youth population and similar economic contexts (Table 1).

**Table 1:** Nigerian tobacco industry and youth keyword search summary using Medline

S/N	Key Words Search	Number of hits	Number of articles downloaded
1	tobacco Industry **AND** target* **AND** youth **OR** adolescents **OR** young people **OR** teen* **OR** young adults **OR** children	153	12
2	Cigarette* **OR** tobacco industry **AND** Nigeria **AND** youth **OR** adolescents **OR** young people **OR** teen* **OR** young adults **OR** children	43	6
3	target* **AND** British American Tobacco **OR** BAT **AND** youth **OR** adolescents **OR** young people **OR** teen* **OR** young adults **OR** children	38	1
4	Tobacco Industry **AND** Nigeria	13	4
5	market* **OR** distribution **AND** tobacco **OR** cigarette* **AND** youth **OR** adolescents **OR** young people **OR** teen* **OR** young adults **OR** children **AND** Nigeria	14	0
6	corporate social responsibility **OR** CSR **AND** tobacco **OR** cigarette* **AND** youth **OR** adolescents **OR** young people **OR** teen* **OR** young adults **OR** children	10	4
7	tobacco Industry **AND** target* **AND** youth **OR** adolescents **OR** young people **OR** teen **OR** young adults **OR** children **AND** Nigeria	1	1
8	Tobacco Industry **AND** smoking initiation **AND** youth **OR** adolescents **OR** young people **OR** teen **OR** young adults **OR** children **AND** Nigeria	0	0
9	tobacco Industry **AND** target* **AND** above the line **OR** ATL **AND** youth **OR** adolescents **OR** young people **OR** teen* **OR** young adults **OR** children	1	1
10	tobacco Industry **AND** target* **AND** below the line or BTL **AND** youth **OR** adolescents **OR** young people **OR** teen* **OR** young adults **OR** children	5	3
	**Total**	**278**	**32**

### Search and analytical strategy

**Fig. 1:**
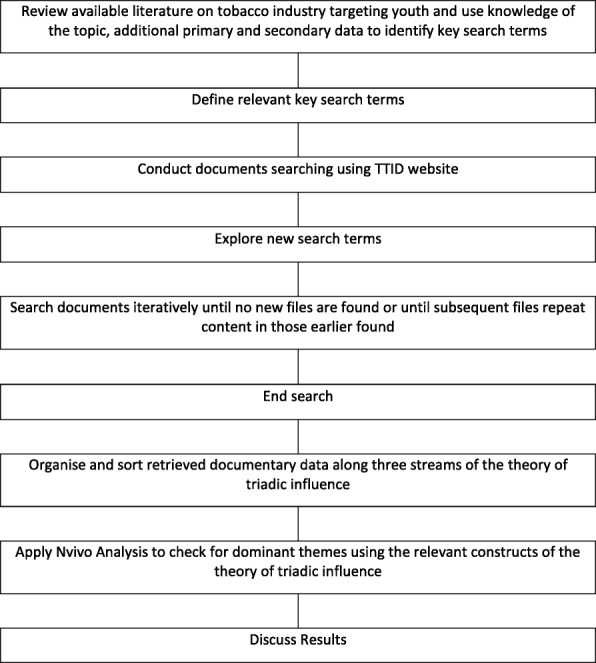
Tobacco Industry Documents (TIDs) search and analysis flowchart

The research systematically searched the Truth Tobacco Industry Documents^1^ database for documents related to tobacco industry targeting of youth in Nigeria (Fig. 1). All hits were reviewed and documents relevant to this research were identified and organized chronologically.

The contents of relevant documents were then coded using Nvivo 11 with themes derived from the three streams of the theory of triadic influence (TTI) concerning youth and tobacco use (Table 2).

**Table 2:** Matrix of types and levels of influence on smoking

Levels of influence	Types of influence		
	Intra-personal (biology/personality)	Interpersonal (Social)	Cultural (Environmental)
**Ultimate**	**Definitions:** Personality traits and intrapersonal characteristics that although beyond the easy control of adolescents, might promote some internal motivation to smoke cigarettes or make them susceptible to the physiological effects of tobacco. **Constructs:** Genetic susceptibility to nicotine; lack of impulse control; external locus of control: aggressiveness; extraversion; sociability; risk-taking; sensation seeking; neuroticism or emotional instability.	**Definitions:** Characteristics of the people who make up adolescents’ most intimate social support system. These characteristics are not specific to smoking and are beyond the personal control of adolescents but nonetheless put them at risk for succumbing to social pressure to smoke. **Constructs:** Infrequent opportunities for rewards from family members; lack of parental warmth, support, or supervision; negative evaluations from parents; home strain; parental divorce or separation; unconventional 'values of parents; unconventional values among peers.	**Definitions:** Aspects of adolescents’ surroundings, neighbourhoods, social institutions, and culture that, although beyond the personal control of adolescents, put them at risk for developing positive attitudes towards tobacco use. **Constructs:** Local crime and employment rates; inadequate schools; poor career and academic options; infrequent opportunities for rewards at school; negative evaluations from teachers; media and advertising depictions of smoking; weak public smoking ordinances; low tobacco taxes; cigarette availability; weak school-level policies on smoking.
**Distal**	**Definitions:** Affective states and general behavioural skills of adolescents that promote some internal motivation to smoke and that undermine their refusal skills. **Constructs:** Low self-esteem; temporary anxiety, stress, or depressed mood; poor coping skills; inadequate social skills; weak academic skills.	**Definitions:** Emotional attachments of adolescents and the tobacco-specific attitudes and behaviours of influential role models who encourage smoking. **Constructs:** Weak attachments to and weak desire to please family members; strong attachment to and strong desire to please peers; greater influence by peers than parents; smoking-specific attitudes and behaviours of role models.	**Definitions:** General values and behaviours of adolescents that contribute to their attitudes toward tobacco use. **Constructs:** Weak commitment to conventional values, school, and religion; social alienation and criticism; weak desire for success and achievement; hedonic values and short-term gratification; rebelliousness; desire for independence from parents; tolerance of deviance.
**Proximal**	**Definitions:** Beliefs about one’s ability to smoke cigarettes and to avoid smoking. **Constructs:** Refusal skills; determination to smoke; use self-efficacy; refuse self-efficacy.	**Definitions:** Beliefs about the normative nature of smoking and pressures to smoke. **Constructs:** Prevalence estimates; motivation to comply with other smokers; beliefs that important others (friends, parents and other role models) encourage smoking.	**Definitions:** Beliefs and evaluations about the costs and benefits of smoking. **Constructs:** Expected costs and benefits of not smoking; evaluation of costs and benefits of not smoking; expected costs and benefits of smoking; attitudes towards smoking by others; attitudes toward smoking by self.
**Immediate predictors**	Decision/intentions Trial behaviour Related behaviours		

Source: Egbe, C.O [12]. *Risk influences for smoking among the youth in Southern Nigeria. PhD Thesis.* University of Kwazulu-Natal. Adapted from Flay, B. R., Petraitis, J., & Hu, F. B [20]. Psychosocial risk and protective factors for adolescent tobacco use. *Nicotine & Tobacco Research*, 1 Suppl 1, S59–65. Retrieved from http://www.ncbi.nlm.nih.gov/pubmed/11072406

### Key informant interviews

To triangulate findings from the literature search and the TIDs, the research conducted key informant interviews with selected policy makers, researchers, media, educationists, and representatives of civil society organizations. The interviews were designed to provide additional information on how the tobacco industry has targeted youth in Nigeria and the current state of tobacco control in Nigeria. Purposive and then snowball sampling were used to identify key informants for semi-structured interviews. Interviewees were recruited using official government and civil society organization’s (CSO) websites, networks and contacts of the supervisory committee and student researcher (first author). A total of 12 interviews were conducted with 6 policy-makers and governmental officials, 2 civil society organization representatives, a high school principal, a journalist and 2 researchers concerned with public health and tobacco control.

### Triangulating data from various sources

**Fig. 2:**
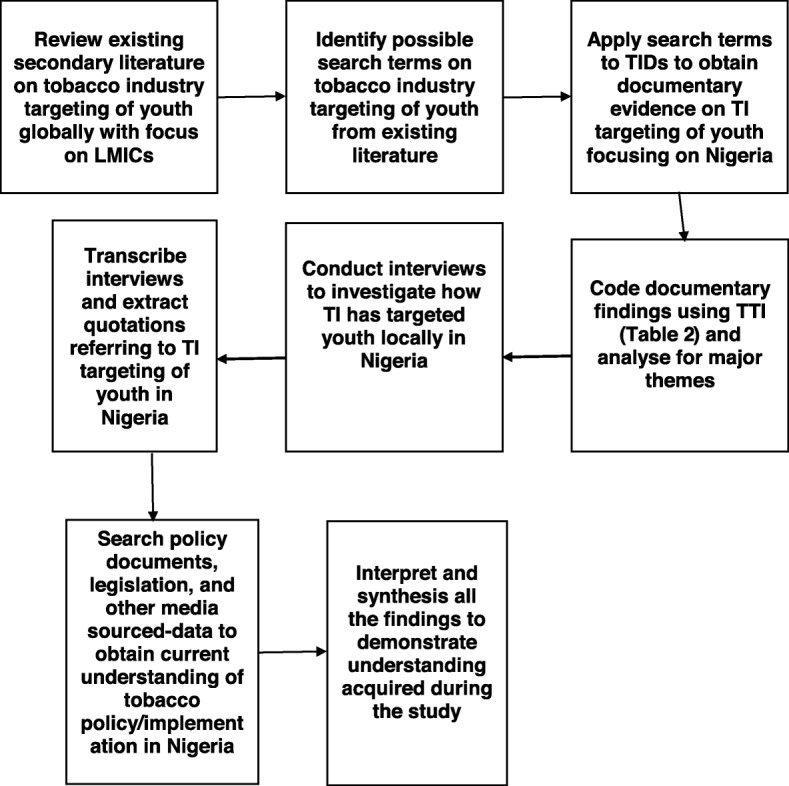
Flowchart for triangulating data from various sources

Figure 2 shows the flowchart for triangulating the data obtained from various sources to develop an understanding of the various issues identified.

## Results

The results below containing quotations from industry sources (TTCs) and their collaborators, mainly market research companies. While market research companies do not constitute a part of the tobacco industry, their opinions can form the basis for tobacco industry marketing activities. A summary of the themes identified in the analysis of the TIDs can be found in Table 3.

**Table 3:** Summary table of themes associated with biology/personality, social, and cultural/environment streams found in this study

Biology/personality	Social	Cultural/environment
Self-image Self-efficacy External Locus of Control Sensation seeking Susceptibility to nicotine	Lack of parental warmth, support, or supervision (leading to ability to hide smoking habit from parents) Greater influence by peers than parents Smoking specific attitudes and behaviours of role models Strong attachment to and strong desire to please peers Beliefs that important others (friends, parents and other role models) encourage smoking Motivation to comply with other smokers	Cigarette availability Media and advertising depictions of smoking Hedonic values and short term gratification Tolerance of deviance Expected costs and benefits of smoking

### Biology/personality stream

- *Self-image:* There is evidence that poor self-image can be a key driver of smoking initiation among youth [25, 27, 31]. This is achieved by associating tobacco use with aspirational messages. The TIDs reviewed suggest that the industry used packaging, above and below the line advertising to portray smokers as successful people, such as wealthy business executives and athletes. One of the brands was said to create a “sparkle” that made young people feel better about themselves. While other brands were seen as fashionable or “brands to be seen with.” Below are some quotes from industry sponsored market research in Nigeria extracted from the TIDs.
  The typical user is envisaged as trendy, rich, but also young - in other words a highly aspirational user image for these smokers (Rodnight, 1991 [40]) .
  Rothmans, a younger brand in this market and generally regarded as an easier smoke, tends to have a younger user image, It seems that it is regarded by younger smokers as the brand to be seen with, as the popular, fashionable choice. Rothmans is more fashionable.
  (Younger, Upper Income, Premium Price, Lagos)" (Market Behaviour Limited, 1991a [28]).
  [The advertising] It’s trying to tell us that, if you take a Benson, it sort of sparkles you, makes you feel yourself.
  (Benson young smoker, Lagos - Nigeria) (Market Behaviour Limited, 1992 [29])

(b) *Self-efficacy:* This refers to confidence in one’s own ability to achieve intended results. The TIDs reviewed suggest that the industry sought to make smoking more attractive to new users by addressing perceptions that smoking behaviour is difficult or problematic. One major challenge, for instance, is the harsh effect of cigarette smoke on the throat of new smokers. The industry thus targeted new smokers in Nigeria with menthol-flavoured cigarettes, such as St. Moritz, to reduce that effect [17, 35], enhancing the confidence of new smokers in using their product, and increasing the likelihood of continued smoking behaviour. An industry sponsored market research quote on Nigeria states:
  The St. Moritz smoker was always spontaneously cited to be young men variously described as 15+, teenagers, youths or beginners. St. Moritz subsequently has something of an image of being a brand for young and inexperienced smokers.
  (Market Trends Limited, 1993c [30])
  The most noticeable now is the flavoured cigarettes that we now have. Once you pick a stick of cigarette, you just want to keep having more. It is naturally enticing to adults, talk less of kids. A youth might pick up the banana flavour and want to experiment. The industry is being very creative in coming up with these ideas.
  (Interview with Philip Jakpor, tobacco control activist, May 6, 2016)
(c) *Sensation-seeking:* This relates to the tendency for youth (notably males) to be particularly attracted to behaviours that involve risk taking. There is evidence from diverse settings that this quest for excitement and adventure among youth is a strong explanatory factor for them becoming new smokers [39, 42]. A desire on the part of youth to try new experiences, deemed exciting and risky, can be used by industry to make the initiation of smoking for the first time more attractive. One of the brands introduced into the Nigerian market to create this effect was the John Player brand as seen in the TID quote below from a market research document on the brand.
  A high quality international brand for the aspiring young and mature smoker who loves leisure. The brand offers rich, full smooth taste.
  (Embagwali, 1992 [14])
(d) *Susceptibility to nicotine:* A more direct effort to influence the biology/personality stream, described in the TIDs reviewed, involved industry efforts to manipulate the nicotine content in cigarettes to optimise their addictiveness. Existing evidence suggests that youth are physiologically more susceptible to nicotine addiction, and more likely to become long-term tobacco users, once addiction is established [5, 11, 36, 42]. Other studies on nicotine addiction show increased tobacco use, by volume and length of time, among people who initiated smoking at a younger age. Research also found quitting behaviour more difficult, given stronger withdrawal symptoms, when initiation is at an earlier age [10, 41]. An industry witness said the following in a Nigerian court about the industry’s attempt to make cigarettes more addictive.
  Addiction was a design criterion of the modern cigarette, achieved through.
  i. manipulation of nicotine levels via technology and blend selection;
  ii. increasing nicotine in the gas phase and/or free nicotine;
  iii. iii decreasing particle size through combustion chemistry;
  iv. increased inhalability through tobacco processing;
  v. specification of flavorants, additives, arid smoke chemistry to promote easy inhalability and thus rapid nicotine absorption;
  vi. development of high-porosity paper, low-pressure drop filtration, rapid burning tobacco, and other characteristics to facilitate rapid and repeated product use; and.
  vii. Marketing, advertising, promotion, and packaging to initiate and sustain addictive use patterns in youth and adults.
  (WA, 2008 [43])

### Social stream

- *Lack of parental warmth, support or supervision:* A TID quote from an industry sponsored market research observes:

Again linked to the overall lightness and mildness of the smoking offer, menthol cigarettes were commended for having very little or no odour. Consequently they do not leave a lingering smell on the body or on clothes which can later be detected by peers or family members (particularly important to younger smokers who know their parents would disapprove of their smoking and who prefer not to upset them by letting than know)".

[(Embagwali, 1992 (Market Trends Limited, 1993c [30])

Previous studies have shown that youth are more likely to smoke if they are able to conceal their habit from their parents. A number of factors could contribute to this including, but not limited to, distrust in parents, lack of parental warmth and love, lack of motivation or encouragement from parents [12, 13, 22]. The tobacco industry strengthens the underage smokers’ ability to hide their smoking status from their parents by providing them with menthol or flavoured cigarettes. Nigeria is a major menthol market [3, 17, 28, 35]. Other countries have controlled youth smoking by banning menthol cigarettes [27, 38]. The expansion of the menthol market in Nigeria thus supports a sustainable increase in underage smokers. Smaller market operators like the International Tobacco Company Limited Nigeria are taking advantage of the menthol and flavoured cigarettes market to boost their sales due to social unacceptability of smoking [17].

(b) *Greater influence by peers than parents:* The influence of peers, in initiating and strengthening smoking habits, is supported by previous research [2, 6, 46]. As observed in an industry sponsored research on Nigeria:
  As in other West African markets, either peer group pressure or the example of parents/older relatives prompts new smokers to try cigarettes.
  (Market Behaviour Limited, 1991a [28])
  Apart from free distribution of cigarettes, since tobacco companies can no longer advertise openly in Nigeria, they do a lot of promotions. They are not going to come out and do big shows like they used to have before, but now they hold secret smoking parties, where young people are used to invite other young people and all this is done in secret and they go to secret locations and they hold parties and cigarettes are distributed for free. (Interview with tobacco control activist, November 21, 2016).

As observed in the quote above, tobacco companies still secretly organise smoking initiation parties to recruit young smokers. These are usually carried out through peer-to-peer invitations due to the illegal nature of the activities. Other themes related to the social stream included: smoking specific attitudes and behaviours of role models, strong attachment to and strong desire to please peers, beliefs that important others (friends, parents and other role models) encourage smoking, and motivation to comply with other smokers.

### Cultural/environment

- *Cigarette availability:* This refers to ease of access and purchase. A marketing plan for Lucky Strike (LSF) brand in Nigeria, made the following observation:

1995 Sales Target

The 1995 total volume of 27.0 million is too high because our strategy is to launch LSF in only six key urban markets. Sales and merchandising activities will be restricted to outlets patronised by YAUS.

(Nigerian Tobacco Company Plc; Ideh, 1995 [34])

**Fig. 3:**
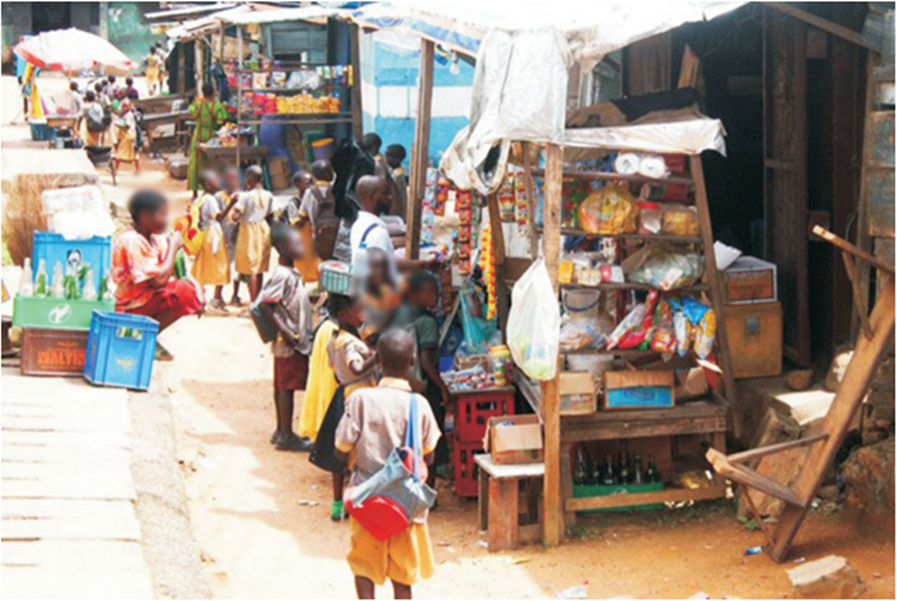
Students buying sweets and biscuits at a kiosk where cigarettes are sold. **Source:** [1]. Big Tobacco Tiny Targets. *Nigeria Tobacco Control Research Group*, 3–45. Retrieved from https://atca-africa.org/pdfs/Big-Tobbacoo-Tiny-Target-Nigeria-Report-Final-press-ready.pdf

The industry clearly noted outlets patronised by young smokers (“YAUS”) and targeted them with brands with a youthful appeal. Figure 3 below shows a “sweet shop” patronised mostly by school children that also sells tobacco products.

**Fig. 4:**
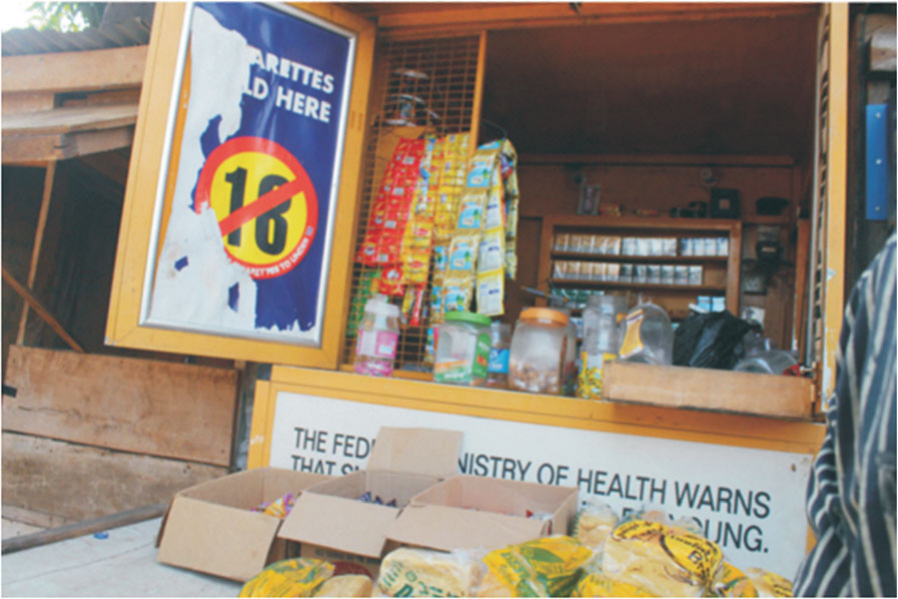
BAT branded shop opposite an International school with the warning sign mutilated and the health warning hidden from view. **Source:** [1]. Big Tobacco Tiny Targets. *Nigeria Tobacco Control Research Group*, 3–45. Retrieved from https://atca-africa.org/pdfs/Big-Tobbacoo-Tiny-Target-Nigeria-Report-Final-press-ready.pdf

**Fig. 5:**
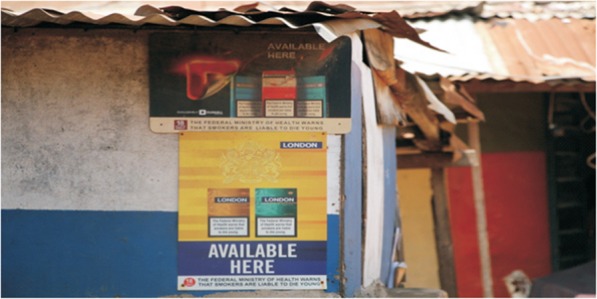
Cigarette advertisement conspicuously pasted on wall. **Source:** [1]. Big Tobacco Tiny Targets. *Nigeria Tobacco Control Research Group*, 3–45. Retrieved from https://atca-africa.org/pdfs/Big-Tobbacoo-Tiny-Target-Nigeria-Report-Final-press-ready.pdf

b) *Media and advertising depictions of smoking*: This refers to hidden and overt messages received by the public when viewing tobacco related ads (see Fig. 4). In the cultural/environment stream it also refers to the extensive use of marketing and advertising to portray smoking in culturally appealing ways. To circumvent advertising restrictions imposed in Nigeria since 1992, the tobacco industry now uses a lot of point-of-sale advertising (See Fig. 5). Another subtle means of advertising is the increased presence of smoking scenes in Nigerian films, many of which are patronised by youth.

c) *Expected costs and benefits of smoking:* This looks at the cost-effectiveness of smoking a specific brand. Cheaper brands tend to sell more among youth because of their lower purchasing power. It also refers to the perceived value that young people derive from smoking cigarettes as shown in the TIDs quotes below.

Silk Cut is sometimes also included because it is recognized as a quality cigarette, although considerably cheaper (50 kobo as opposed to N1 per stick). It is generally seen as a milder version of RKS, of particular interest to younger smokers (mildness and low price) …

(Market Behaviour Limited, 1992 [29])

“‘Discover Gold’. Gold is something everybody wants to own - that is their marketing strategy.” (Rothmans young smoker, Lagos - Nigeria).

(Market Behaviour Limited, 1992 [29])

The research identified other major themes in cultural/environment stream including hedonic values and short term gratification, which refers to the immediate perceived benefits of tobacco use especially among youth, and tolerance of deviance, which indicates a rebellious mindset ready to be different, for example a teenager who smokes to spite his or her non-smoking parents.

## Discussion

The findings of this research suggest that the tobacco industry has been targeting youth in Nigeria since the 1990s as an emerging market. The limited evidence available suggests that tobacco use among youth in Nigeria remains relatively low, despite these efforts, compared to some emerging markets. The Nigerian federal and state governments appear to have an important opportunity to prevent an increase in youth tobacco use, and thus avoid the substantial burden from tobacco-related disease and death experienced in other emerging markets such as China, India and in the Middle East.

In relation to the biology/personality stream of the theory of triadic influence, self-efficacy and refusal skills appeared to be a major influence on young smokers’ trial and eventual addiction to nicotine. Results of tobacco-industry sponsored market research suggest that the self-efficacy of the smoker was targeted by the marketing of menthol cigarettes, making it easier for a young smoker to start and continue smoking cigarettes. Other major channels of influence targeted by the tobacco industry were media depictions of cigarette brands, pricing and availability (cultural/environment stream).

This research found evidence that the industry targeted factors under the social stream of the theory of triadic influence, namely the ability of young smokers to hide their smoking habits from parents and approval of peers. Ease of access by youth to tobacco products was enhanced by retailers who engaged in underage sales, selling single sticks and retail outlets near schools and other locations where youth frequent. Sales near schools means students can sustain their smoking habits while in school without ever having to carry a stick of cigarrete home. The sales in single sticks further enhances the affordability of the product. While there are some regulations restricting sales to minors, there appears to be an absence of monitoring and enforcement by relevant authorities at the federal, state and local government levels. FCTC Article 16 addresses sales of tobacco products to and by minors [32].

This research found evidence of the targeting of youth by the tobacco industry through CSR activities. Nigeria currently permits the tobacco industry to engage in YSP programmes, such as industry-sponsored YSP messages communicated at the point of sale. This is despite the FCTC Article 5.3 and the NTCA prohibiting all forms of CSR by the industry. This research found that the limited contemporary evidence of tobacco industry activities targeting youth in Nigeria comes from scholars, civil society organizations and media reports of their findings. In view of the slow pace of implementation of the NTCA, these groups have disseminated information to the public on the weak state of tobacco control in Nigeria and the links between government and industry. CSOs have also strengthened implementation of the FCTC.

## Conclusion

In summary, the theory of triadic influence offers a useful analytical framework for categorising factors that influence youth smoking initiation and the associated strategies of the tobacco industry targeting youth in Nigeria. As earlier noted, the industry did not conceptualize its activities within the framework outlined by Flay. However the theory provides a valuable approach to understand the mechanisms by which TTCs have attempted to influence youth smoking behaviour.

The data obtained from the TID search showed that the tobacco industry strategies and activities were associated with factors in all three streams of the theory of triadic influence, notably the cultural/environment stream. A number of dominant themes arise under each stream through this analysis; notably, use of media and advertising to depict smoking behaviours in aspirational ways; ensuring product availability in venues frequented by youth; addressing perceived cost and benefits of smoking (cultural/environment); messaging to overcome social stigma based on a desire to conform with peers (social); and adopting strategies to enhance self-image through tobacco use behaviours (biology/personality). Using the theory of triadic influence can however limit industry strategies identified as the theory was designed to study behaviour change. As a result those strategies that are not directly related to behaviour change or smoking initiation may not have been identified in this research.

### Suggestions for improving tobacco control in Nigeria

Based on the findings of this research, the following suggestions are put forward for strengthening tobacco control policies to counter the targeting of youth by the tobacco industry:

i) The Nigerian federal government signed the National Tobacco Control Act 2015. This act has the potential to prevent a tobacco epidemic among youth if well implemented. The authors suggest the Federal Government of Nigeria and its agencies prioritize the implementation of this act as a tool for enhancing tobacco control within Nigeria.
ii) The Federal Government of Nigeria has already taken steps to investigate the effects of menthol in cigarettes [4]. New regulations banning production, sale or importation of menthol and other flavoured cigarettes would be a strong measure to curb youth smoking in Nigeria.
iii) The National Tobacco Control Act clearly bans smoking in primary and secondary schools. To further strengthen this regulation, the government can enact regulations banning all cigarette sales near primary and secondary schools to reduce the ability of the tobacco industry to shape factors within the cultural/environmental stream that encourage youth tobacco consumption
iv) Considering the interest of the tobacco industry in youths of school age, the Nigerian government can expand school-based cessation measures, taking into account social factors in their design and implementation. Currently no school-based cessation programs exist on a national scale in Nigeria (and in many LMICs).
v) The government can implement measures, including new legislation, to prevent industry interference in shaping public health policies based on FCTC Article 5.3 guidelines, including banning all forms of corporate social responsiility (CSR) activities purporting to support youth smoking prevention.
vi) The government can increase its support for civil society anti-tobacco organizations to enable them to become effective partners in monitoring tobacco industry activities.
vii) The government can allocate significant financial resources to expanding its research capacity to enable it to monitor the industry more effectively, including the marketing of tobacco to youth.

## Data Availability

The data that support the findings of this study are openly available in the Truth Tobacco Industry Documents at https://www.industrydocumentslibrary.ucsf.edu/tobacco. Interview data that support the findings of this study are available from the authors, upon reasonable request.

## Footnotes

1 In 1998, about six million formerly confidential documents from seven tobacco companies and their affiliated institutions became publicly available as a result of successful litigation by former smokers in several states across the US [23]
